# Supplementary material for: Isoquinolinequinone Derivatives from a Marine Sponge (Haliclona sp.) Regulate Inflammation in In Vitro System of Intestine
Source: Mar Drugs. 2021 Feb 4;19(2):90. doi: 10.3390/md19020090 (PMC7913985; doi:10.3390/md19020090)
Supplement: Supplementary file 1 [file marinedrugs-19-00090-s001.pdf]

marine drugs

Supplementary Materials

Isoquinolinequinone derivatives from a marine sponge (Haliclona sp.) regulate intestinal inflammation

Yun Na Kim[1†], Yeong Kwang Ji[2†], Na-Hyun Kim[3], Nguyen Van Tu[4], Jung-Rae Rho[2*] and Eun Ju Jeong[1*]

[1] Department of Agronomy and Medicinal Plant Resources, Gyeongnam National University of Science and Technology, Jinju 52725, Republic of Korea
[2] Department of Oceanography, Kunsan National University, Gunsan 54150, Republic of Korea
[3] Gyeongnam Department of Environment & Toxicology, Korea Institute of Toxicology, 17 Jegok-gil, Munsan-eup 52834, Republic of Korea
[4] Institute of Tropical Biology, 85 Tran Quoc Toan Street District 3, Ho Chi Minh, Vietnam
* Correspondence: jrrho@kunsan.ac.kr(J.R.); Tel.: +82 63 469 4606(J.R.); ejjeong@gntech.ac.kr(E.J.J); Tel.: +82 55 751 3224 (E.J.J.)

Contents

Table S1. Spectral data for compound **8** in $CDCl_3$ (500Hz, for ^1H; 125 Hz for ^{13}C). 13

Table S2. Coordinate for the optimized conformer of compound **1**. 20

Table S3. Experimental and calculated ^{13}C NMR chemical shifts of compound **1**. 21

Figure S1. HRqTOFMS for **1**. 3

Figure S2. ^1H NMR (500 MHz, $CDCl_3$) spectrum of **1**. 4

Figure S3. ^{13}C NMR (125 MHz, $CDCl_3$) spectrum of **1**. 4

Figure S4. HSQC NMR (500 MHz, $CDCl_3$) spectrum of **1**. 5

Figure S5. HMBC NMR (500 MHz, $CDCl_3$) spectrum of **1**. 5

Figure S6. HRqTOFMS for **2**. 6

Figure S7. ^1H NMR (500 MHz, $CDCl_3$) spectrum of **2**. 7

Figure S8. ^{13}C NMR (125 MHz, $CDCl_3$) spectrum of **2**. 7

Figure S9. HSQC NMR (500 MHz, $CDCl_3$) spectrum of **2**. 8

Figure S10. HMBC NMR (500 MHz, $CDCl_3$) spectrum of **2**. 8

Figure S11. HRqTOFMS for **3**. 9

Figure S12. ^1H NMR (500 MHz, $CDCl_3$) spectra of **3**(a) and **4**(b). 10

Figure S13. ^{13}C NMR (125 MHz, $CDCl_3$) spectra of **3**(a) and **4**(b). 11

Figure S14. HRqTOFMS for **8**. 12

Figure S15. ^1H NMR (500 MHz, $CDCl_3$) spectrum of **8**. 14

Figure S16. ^{13}C NMR (125 MHz, $CDCl_3$) spectrum of **8**. 14

Figure S17. COSY NMR (500 MHz, $CDCl_3$) spectrum of **8**. 15

Figure S18. HSQC NMR (500 MHz, $CDCl_3$) spectrum of **8**. 16

Figure S19. HMBC NMR (500 MHz, $CDCl_3$) spectrum of **8**. 16

Figure S20. NOESY NMR (500 MHz, $CDCl_3$) spectrum of **8**. 17

Figure S21. Cytotoxicity of compounds **1~8** against THP-1 cells. 18

Figure S22. Cytotoxicity of compounds **1~8** against Caco-2 cells. 18

Figure S23. In vitro co-culture system of Caco-2 and THP-1 macrophages 19

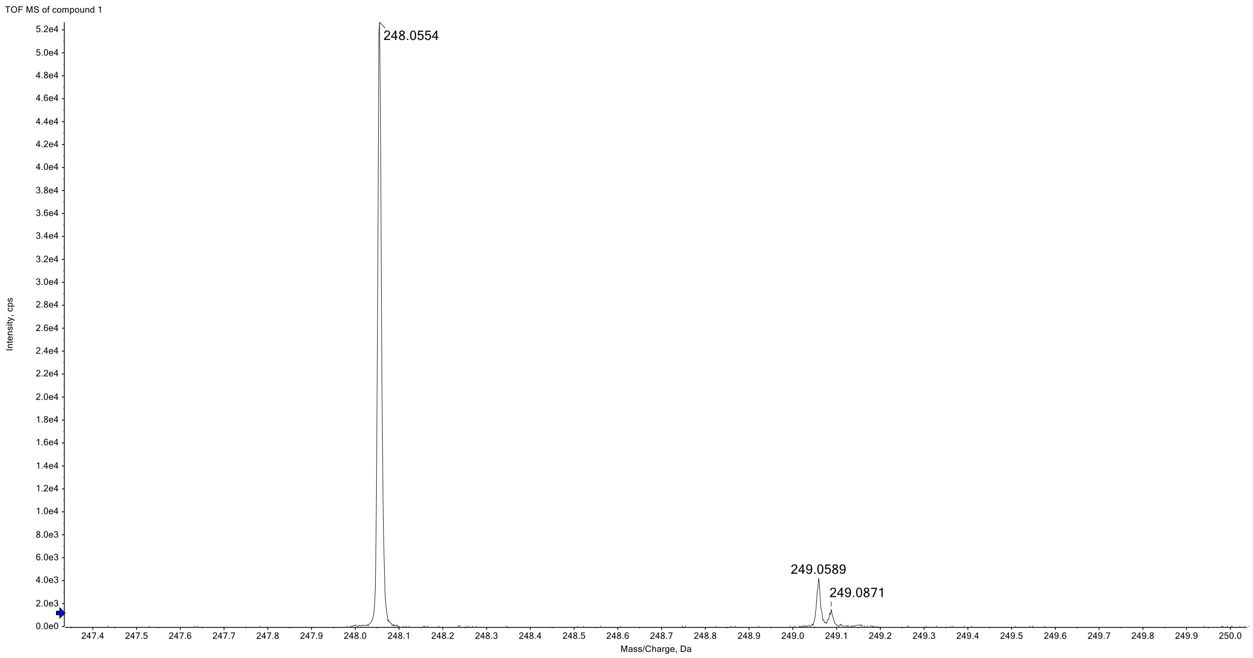

Figure S1. HRqTOFMS for **1**.

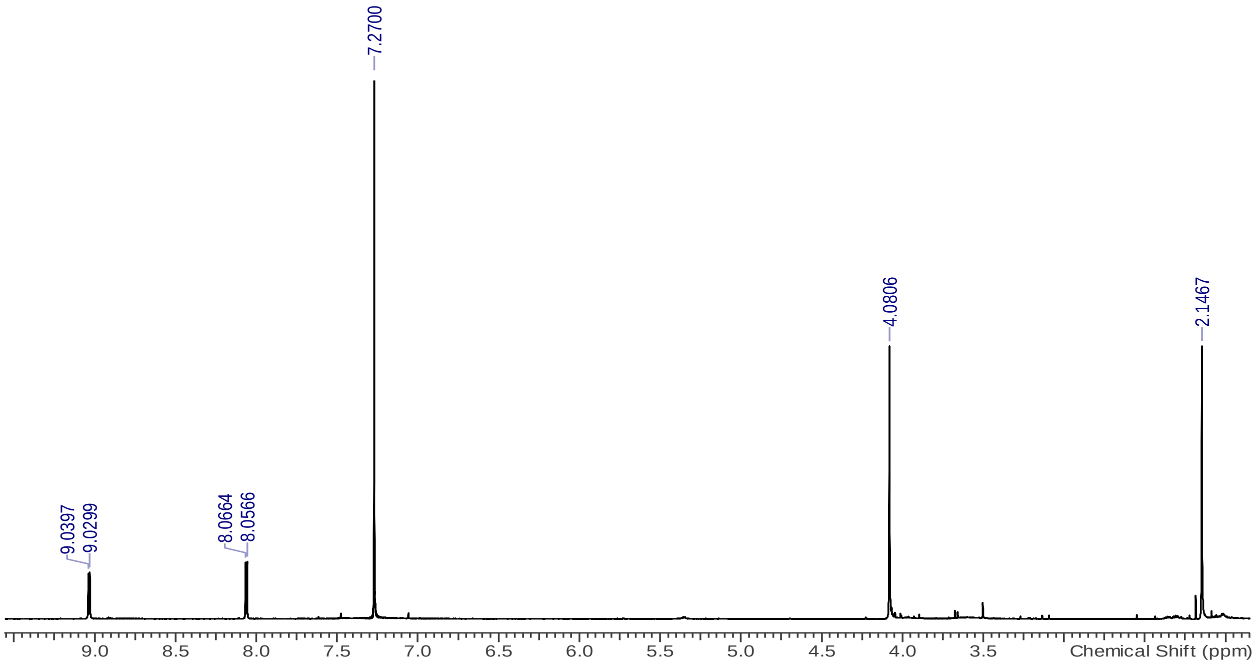

Figure S2. ^{1}H NMR (500 MHz, $CDCl_3$) spectrum of **1**.

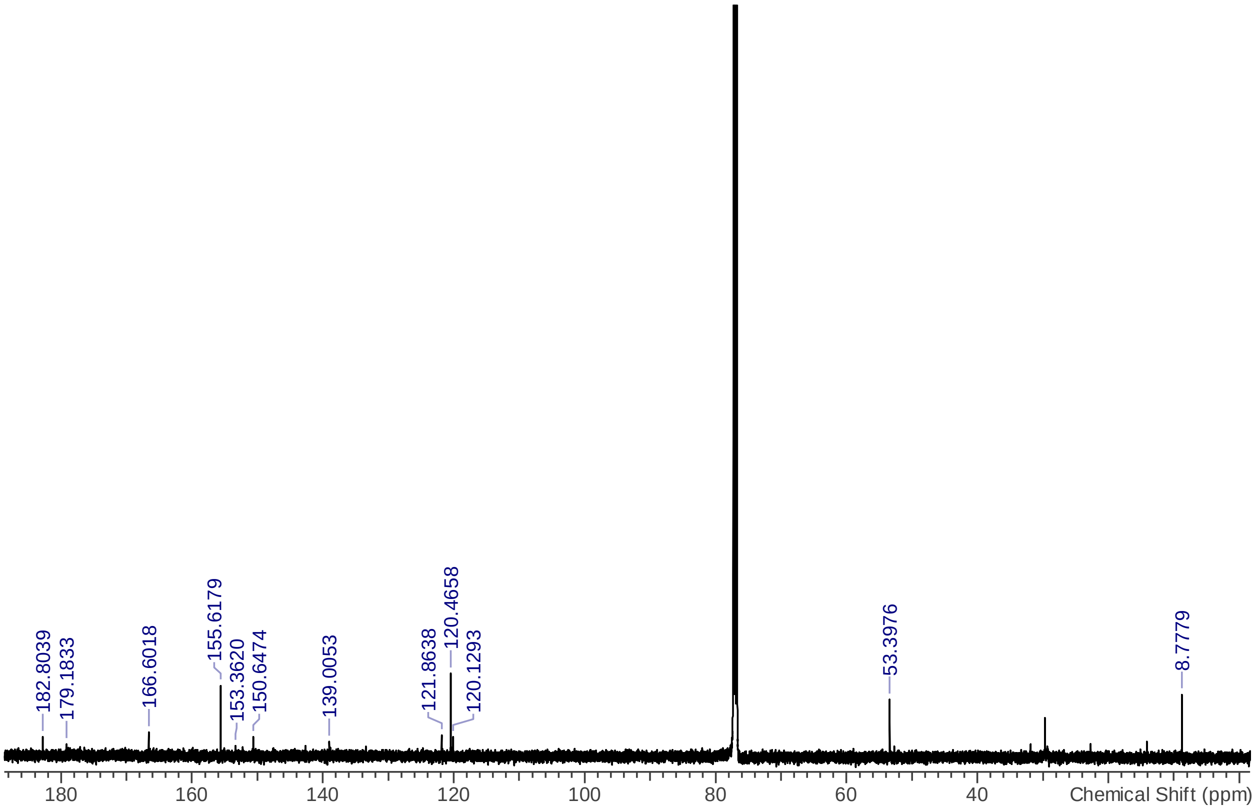

Figure S3. ^{13}C NMR (125 MHz, $CDCl_3$) spectrum of **1**.

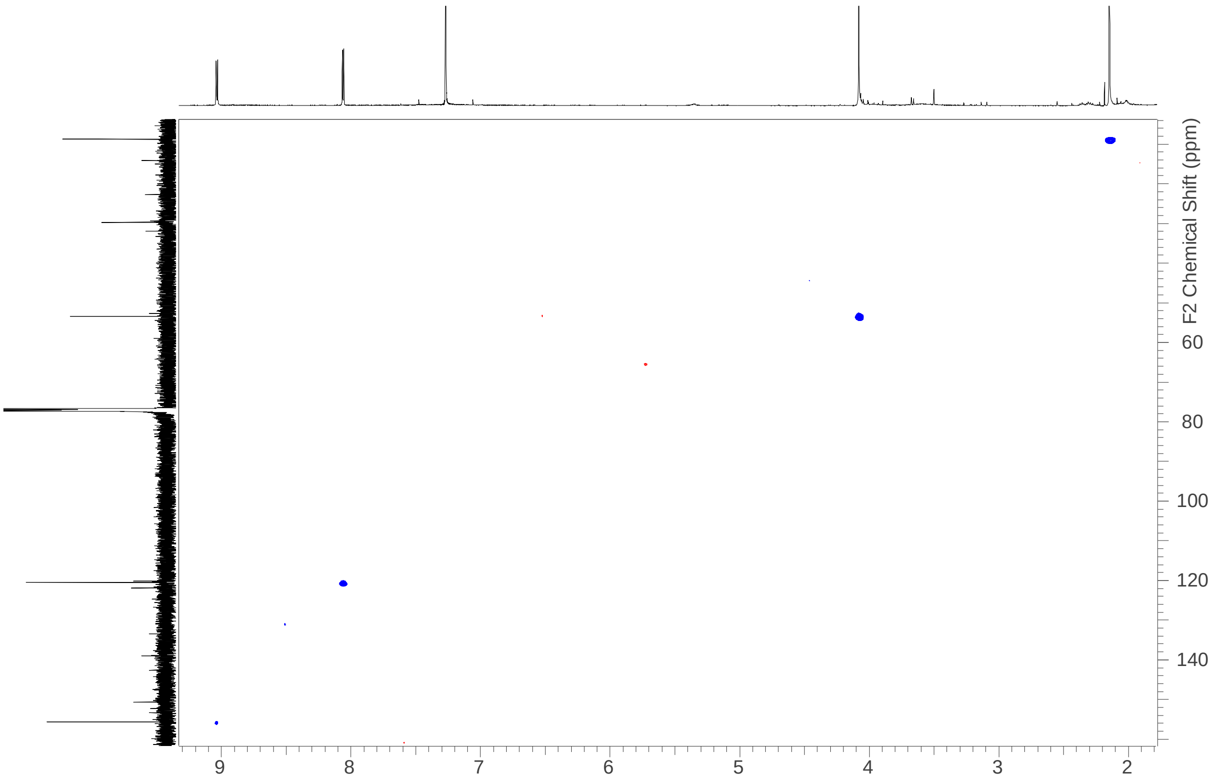

Figure S4. HSQC NMR (500 MHz, $CDCl_3$) spectrum of **1**.

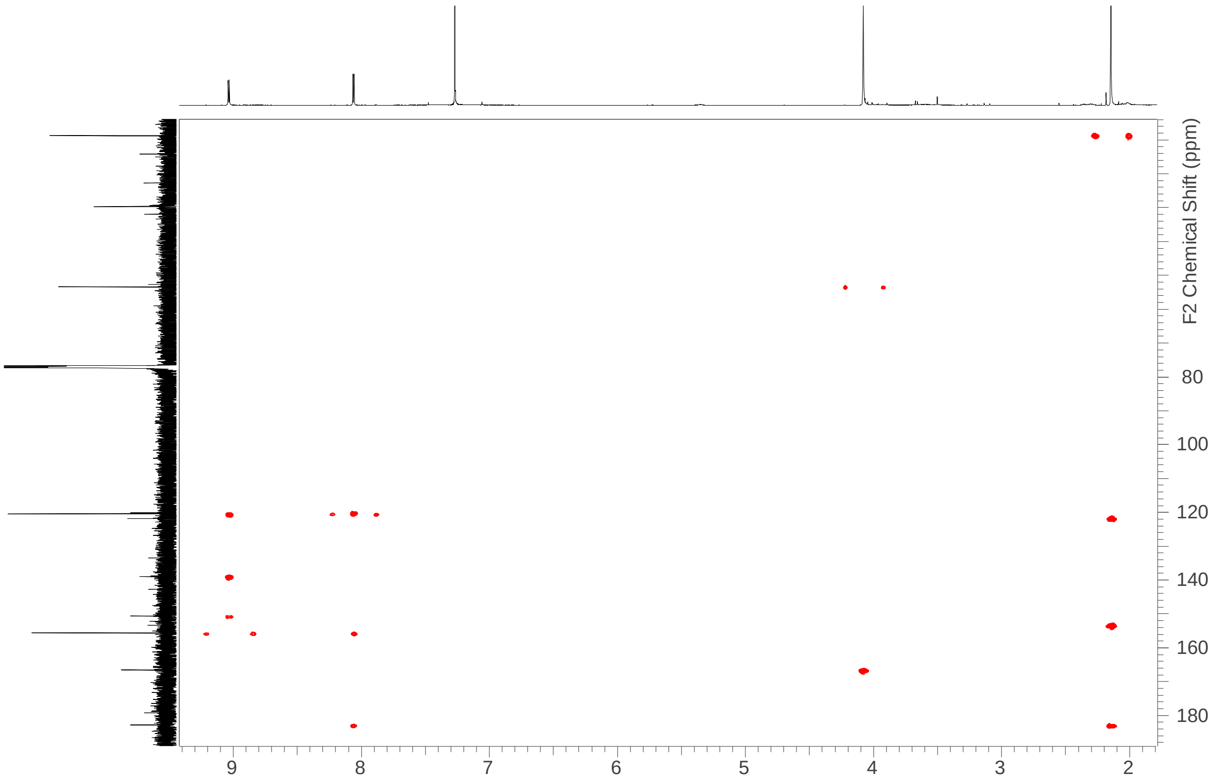

Figure S5. HMBC NMR (500 MHz, $CDCl_3$) spectrum of **1**.

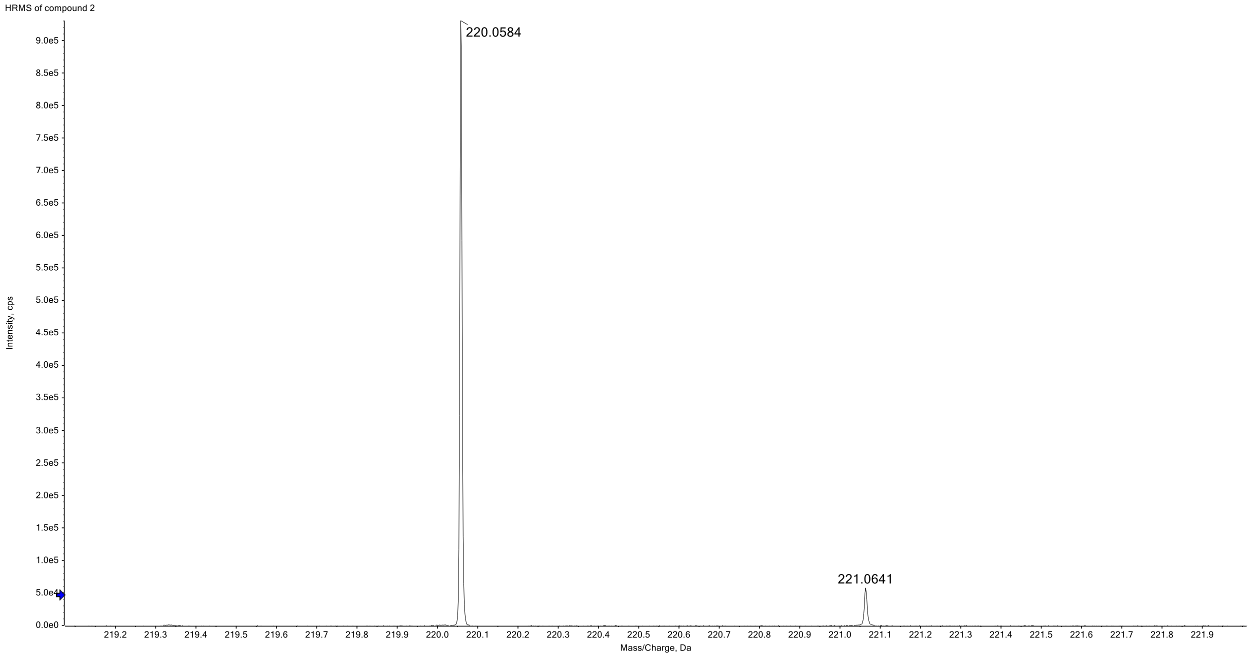

Figure S6. HRqTOFMS for **2**.

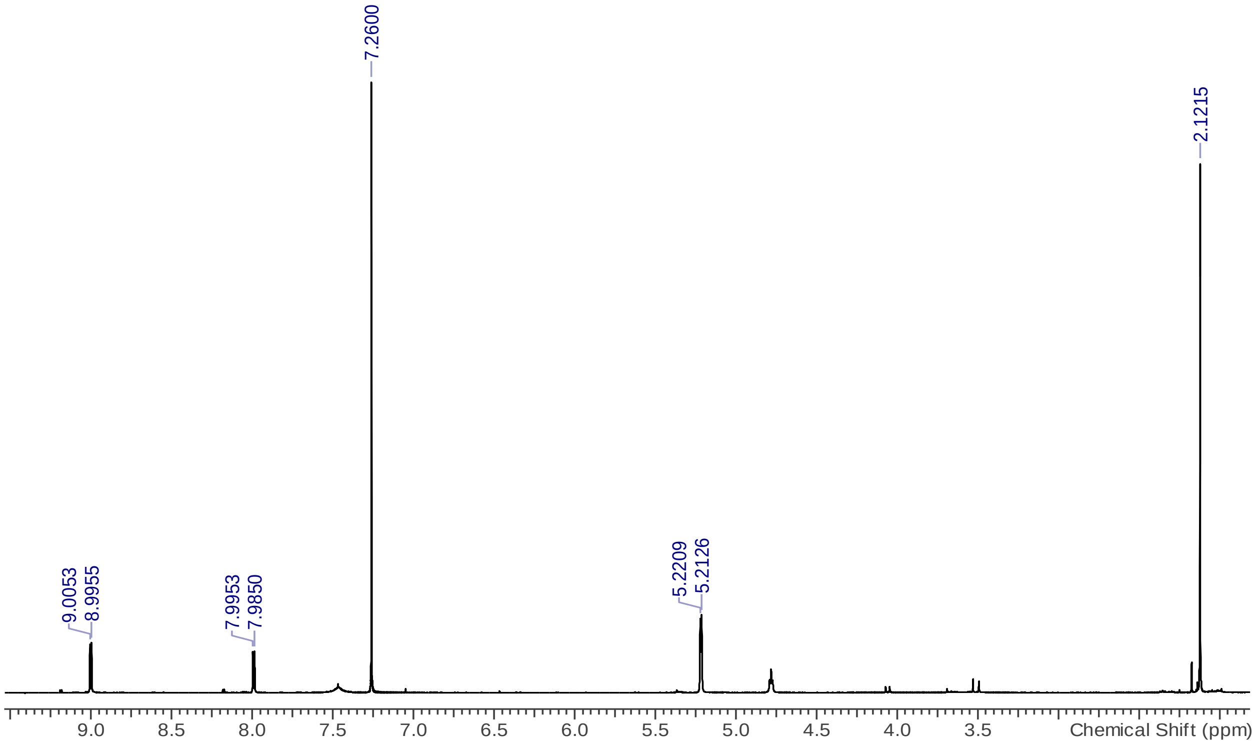

Figure S7. ^{1}H NMR (500 MHz, $CDCl_3$) spectrum of **2**.

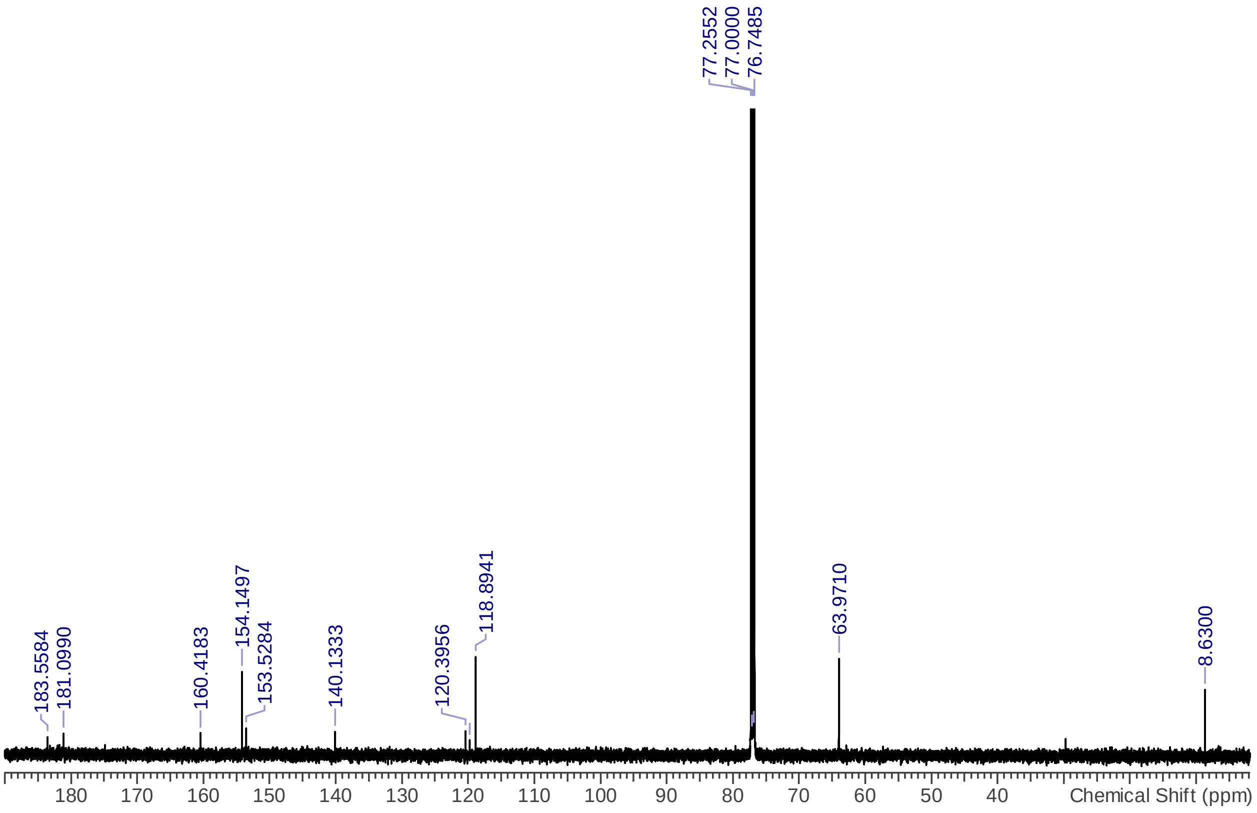

Figure S8. ^{13}C NMR (125 MHz, $CDCl_3$) spectrum of **2**.

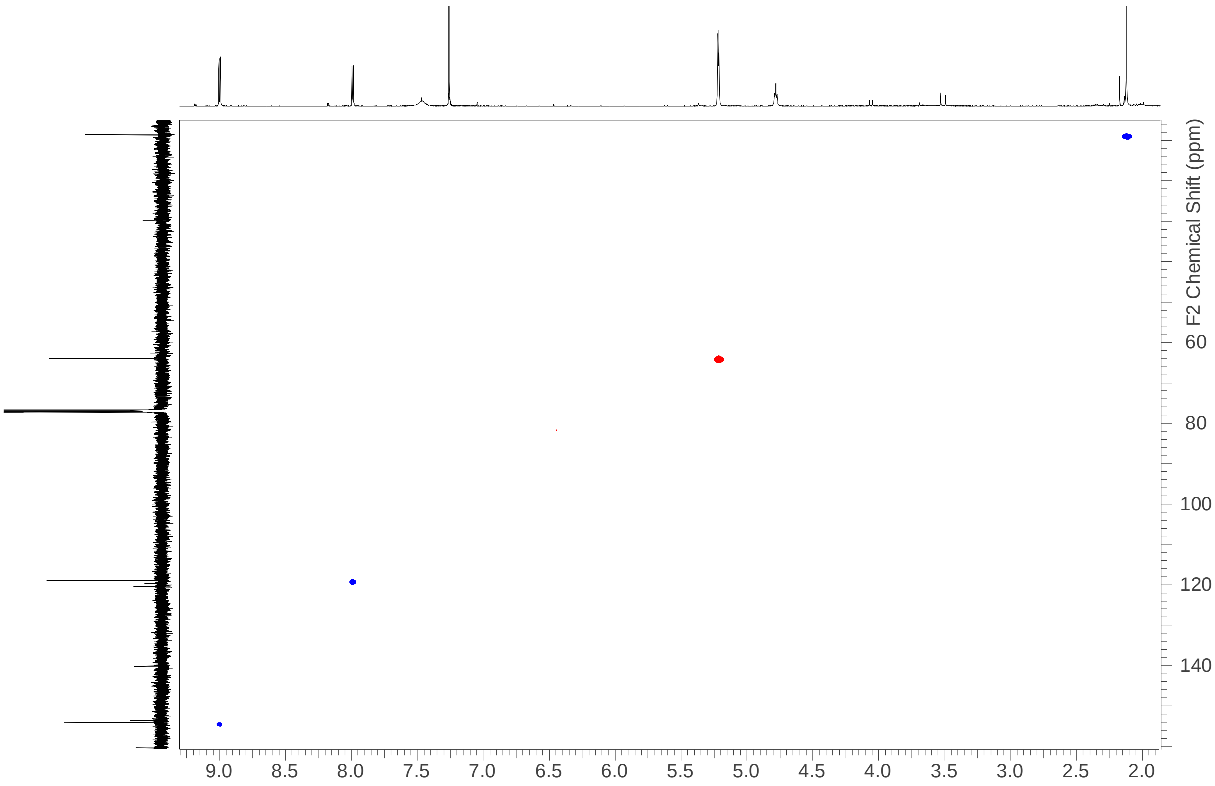

Figure S9. HSQC NMR (500 MHz, $CDCl_3$) spectrum of **2**.

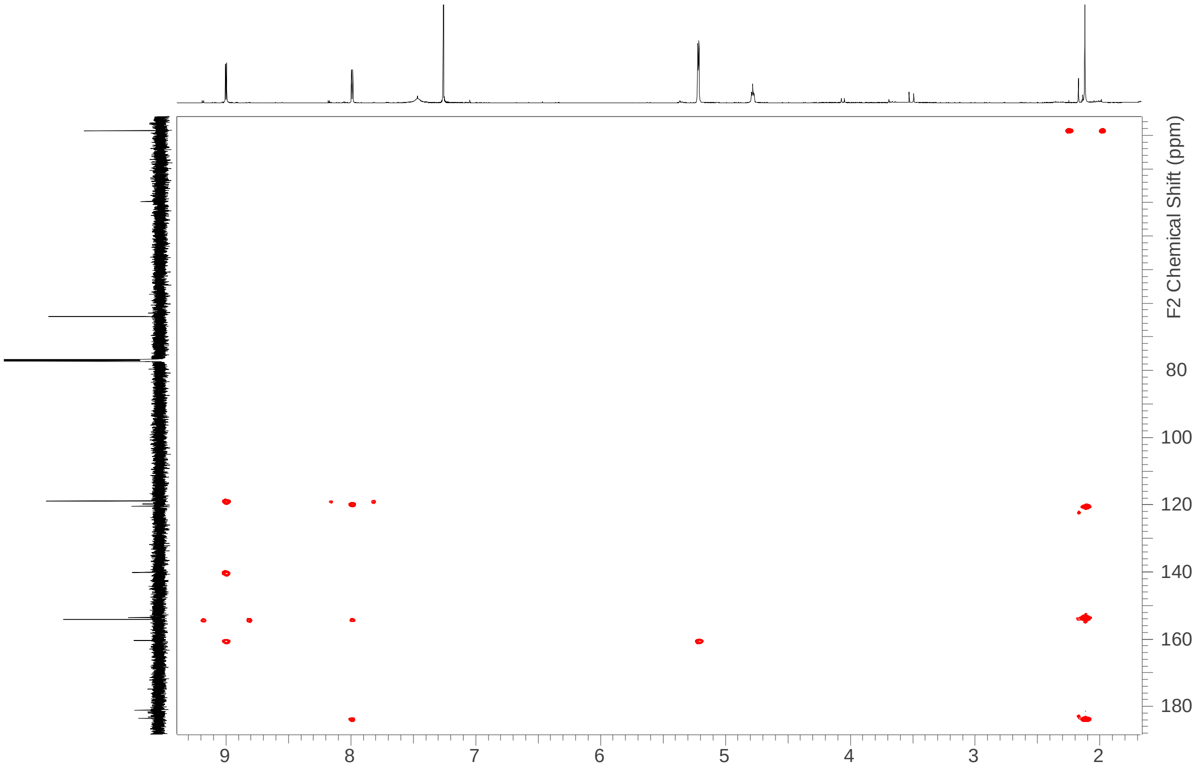

Figure S10. HMBC NMR (500 MHz, $CDCl_3$) spectrum of **2**.

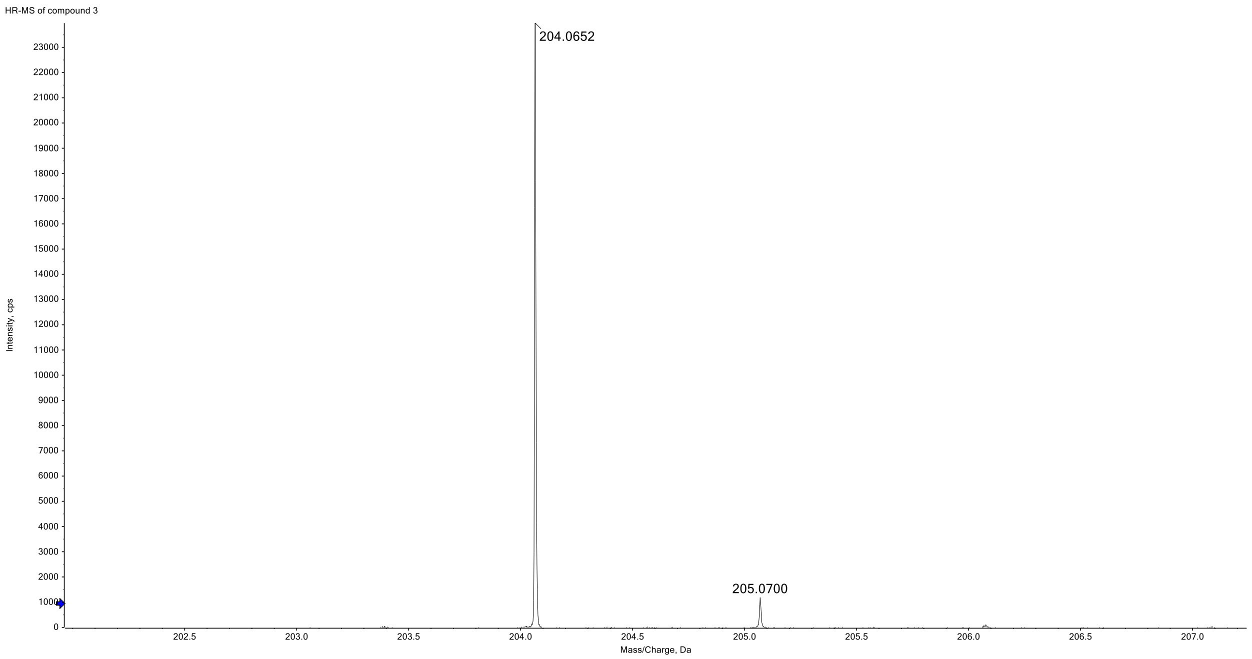

Figure S11. HRqTOFMS for **3**.

(a)

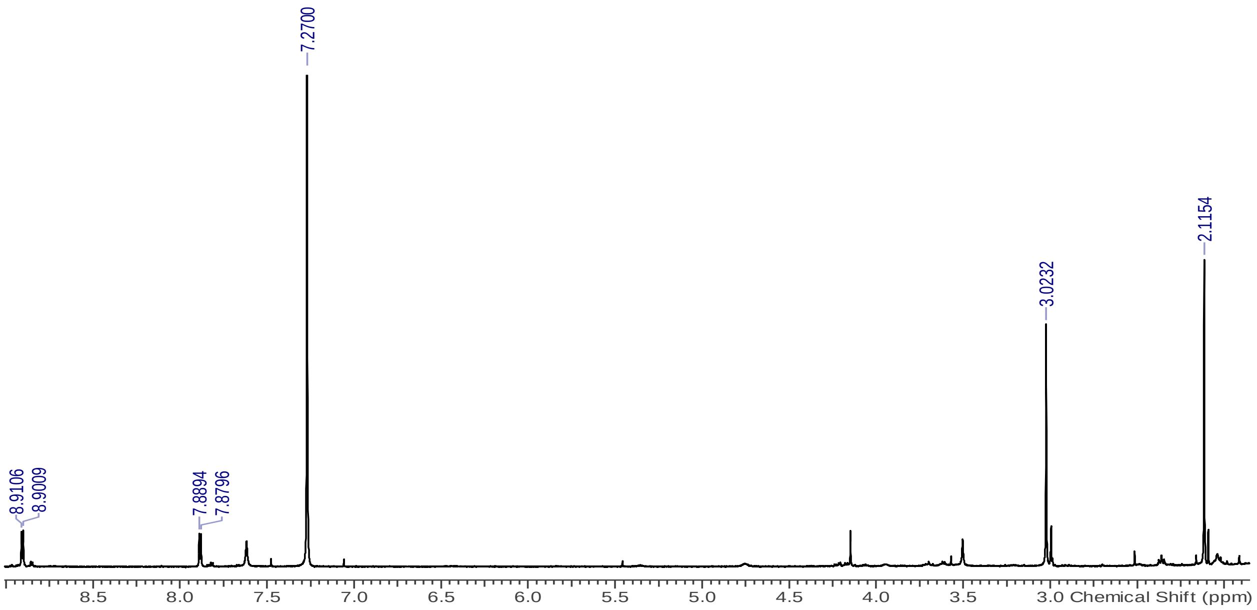

(b)

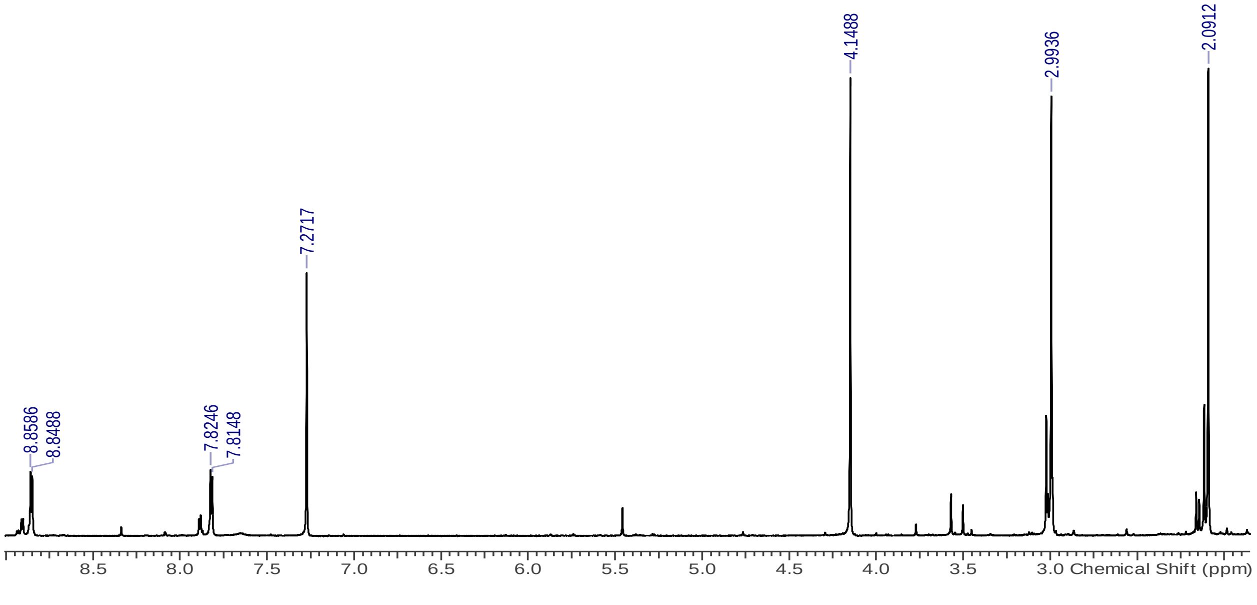

Figure S12. ^{1}H NMR (500 MHz, $CDCl_3$) spectra of **3**(a) and **4**(b).

(a)

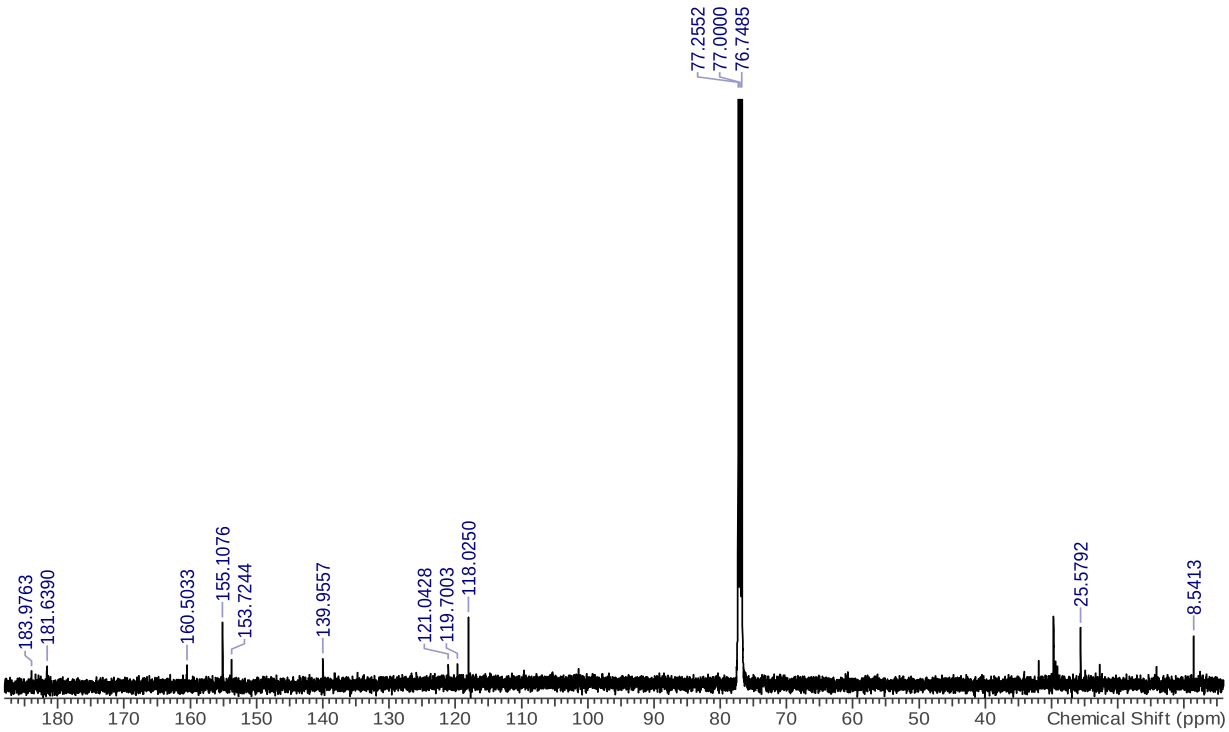

77.2552
77.0000
76.7485
183.9763
181.6390
160.5033
155.1076
153.7244
139.9557
121.0428
119.7003
118.0250
25.5792
8.5413
180
170
160
150
140
130
120
110
100
90
80
70
60
50
40
Chemical Shift (ppm)

(b)

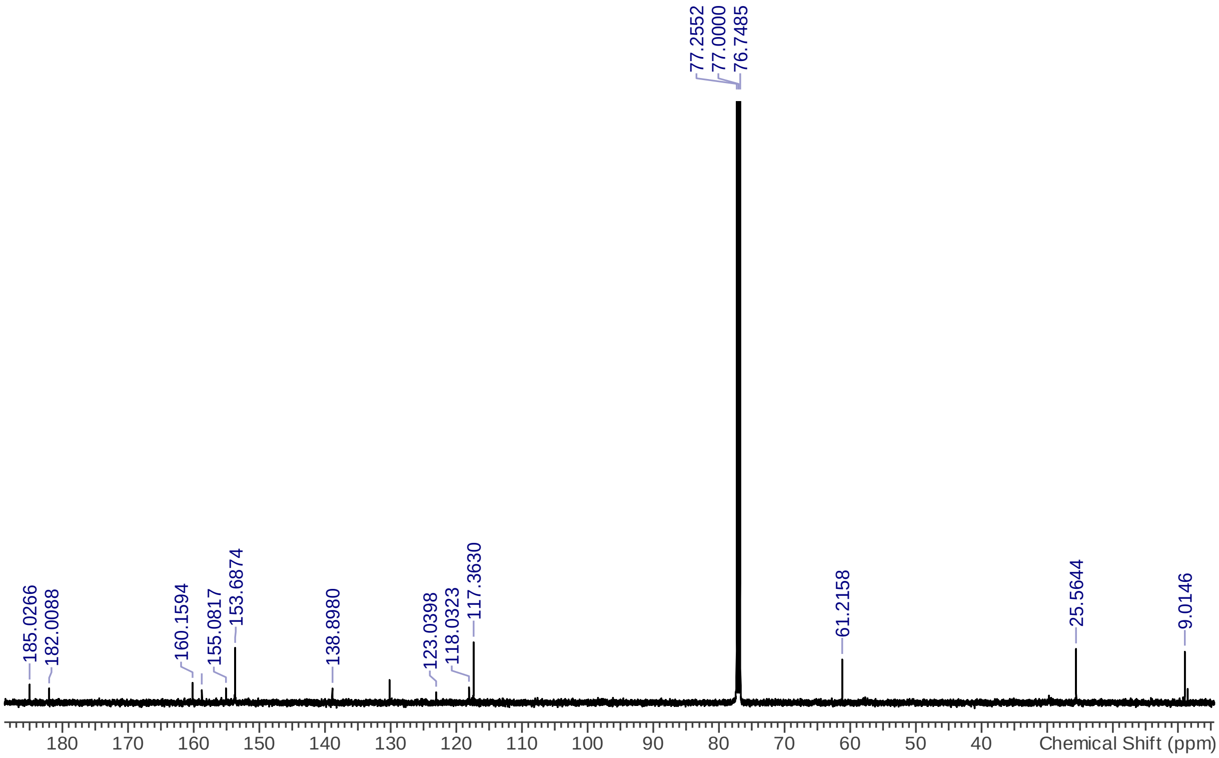

Figure S13. ^{13}C NMR (125 MHz, $CDCl_3$) spectra of **3**(a) and **4**(b).

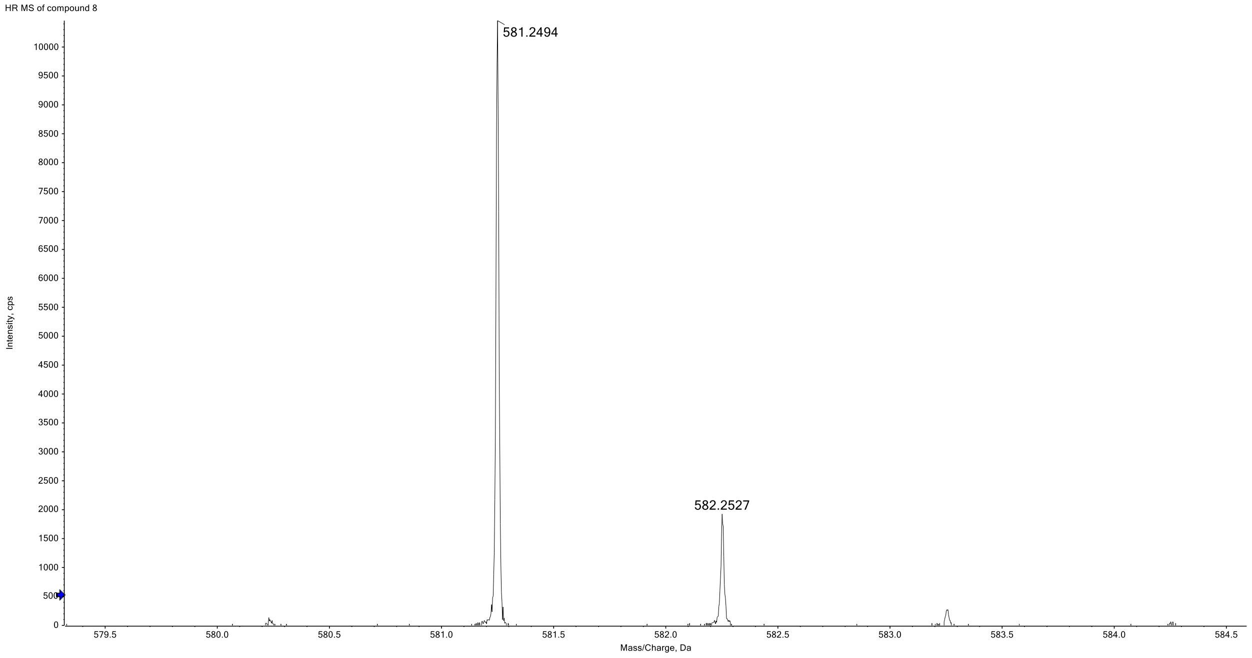

Figure S14. HRqTOFMS for **8**.

Table S1. Spectral data for compound **8** in $CDCl_3$ (500Hz, for ^1H; 125 Hz for ^{13}C)

no	$\delta^{13}C$, mult	δH, mult(*J* Hz)
1	58.4, CH	3.62, m
3	56.0, CH	2.63, dt(11.4, 2.6)
4	25.8, CH_2	1.22, ddd(11.4, 11.4, 3.7); 2.74, dd(11.4, 2.6)
5	185.7, C	
6	129.7, C	
7	155.1, C	
8	181.5, C	
9	136.9, C	
10	141.62, C	
11	55.1, CH	4.04, br d(2.6)
13	57.7, CH	3.23, m
14	71.8, CH	3.89, br s
15	185.7, C	
16	128.0, C	
17	156.1, C	
18	183.2, C	
19	136.2, C	
20	141.58, C	
21	56.1, CH_2	2.77, dd(11.0, 3.2); 3.11, dd(11.0, 2.2)
22	62.8, CH_2	4.26, dd(11.4, 2.5); 4.32, dd(11.4, 2.9)

24	167.0, C	
25	126.7, C	
26	139.4, CH	5.93, qq(7.2,1.4)
27	15.6, CH_3	1.79, dq(7.2, 1.4)
6-CH_3	8.67, CH_3	1.91, s
16-CH_3	8.65, CH_3	1.94, s
25-CH_3	20.3, CH_3	1.57, br s
7-OCH_3	60.9, CH_3	3.96, s
14-OCH_3	59.2, CH_3	3.54, s
17-OCH_3	60.8, CH_3	4.00, s
12-NCH_3	42.1, CH_3	2.46, s

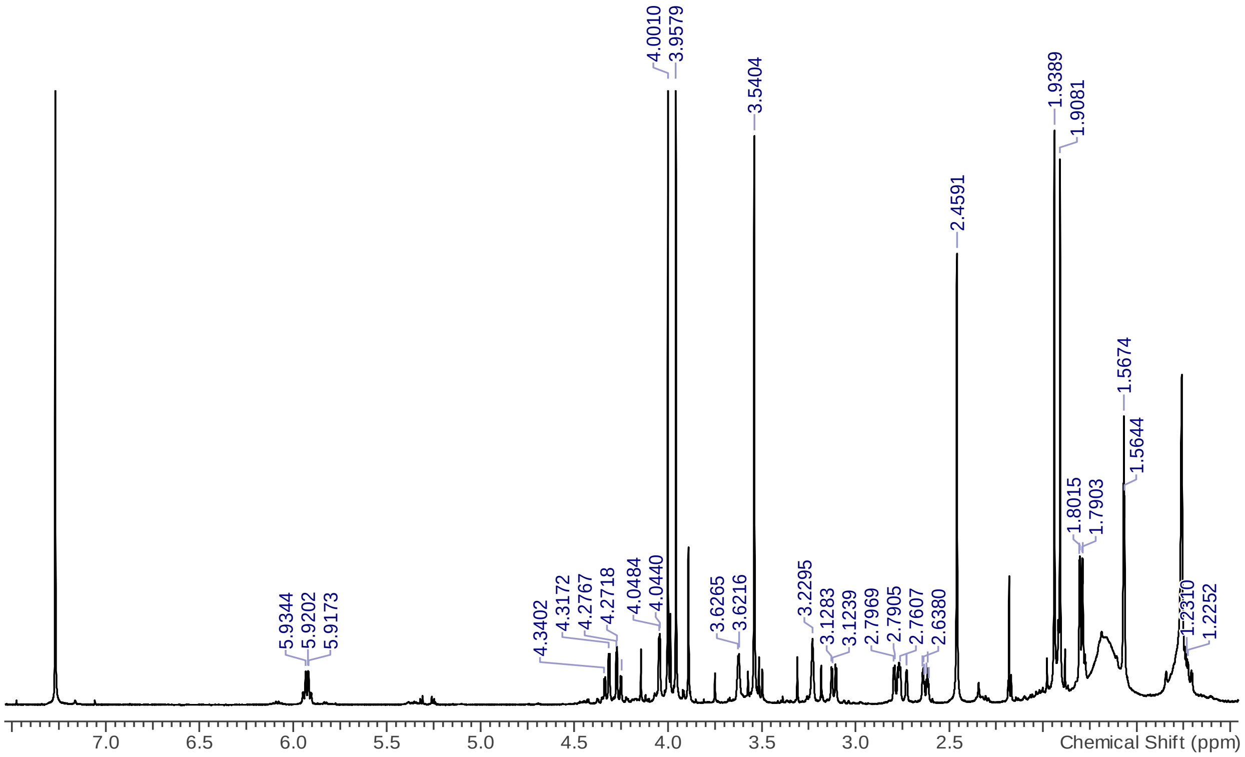

Figure S15. ^{1}H NMR (500 MHz, $CDCl_3$) spectrum of **8**.

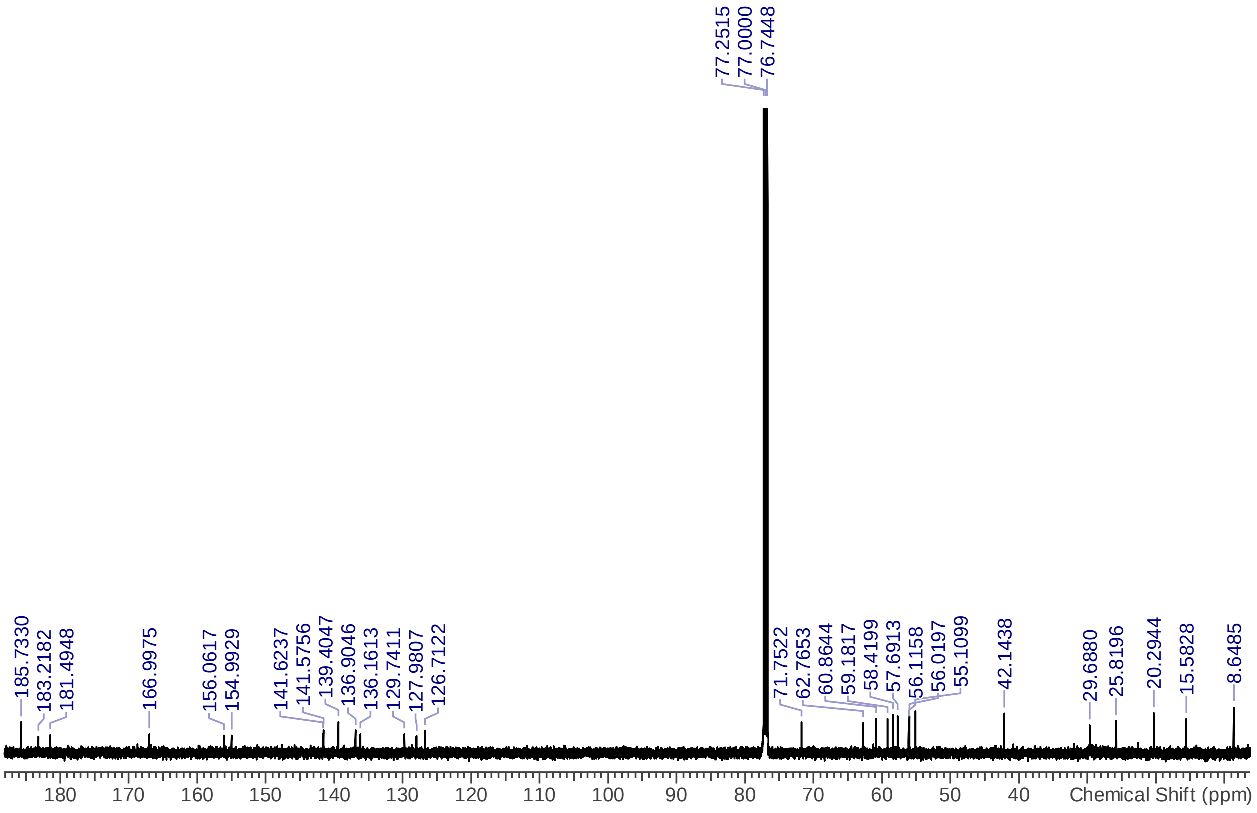

Figure S16. ^{13}C NMR (125 MHz, $CDCl_3$) spectrum of **8**.

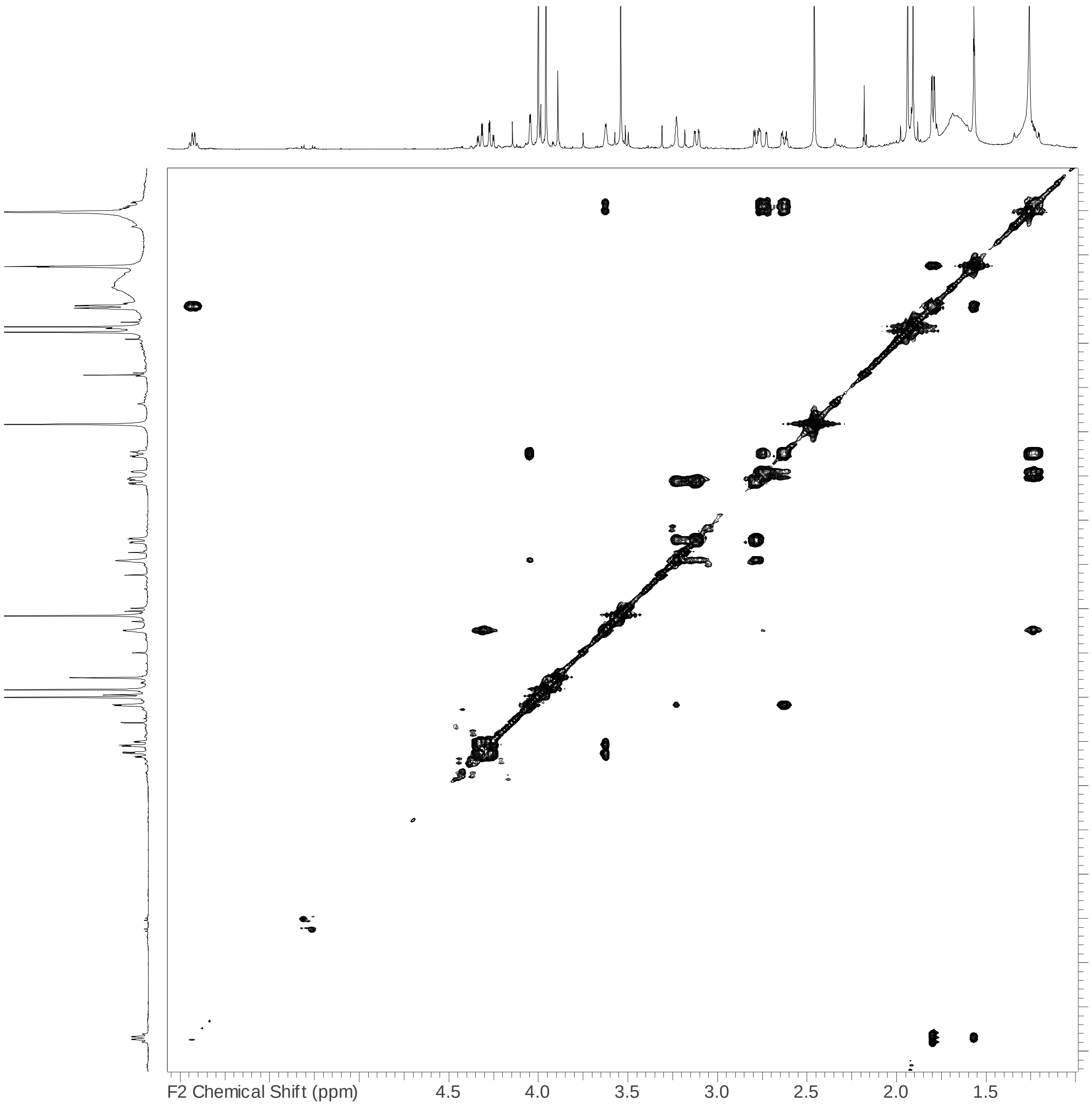

Figure S17. COSY NMR (500 MHz, $CDCl_3$) spectrum of **8**.

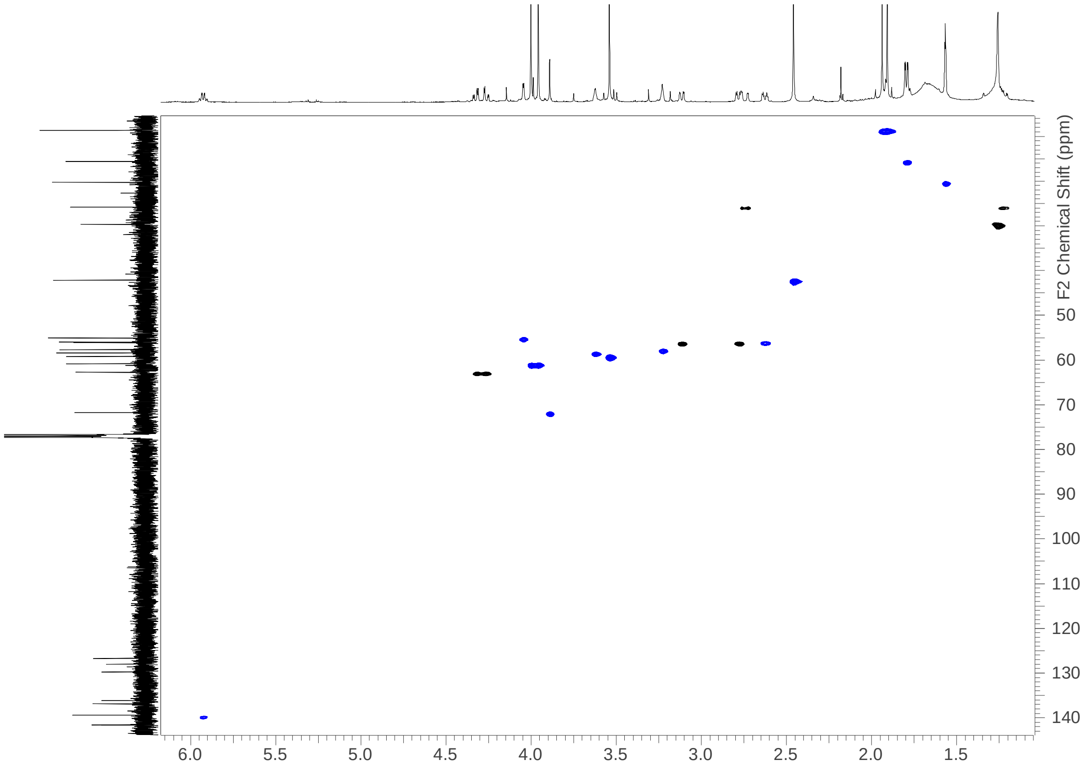

Figure S18. HSQC NMR (500 MHz, $CDCl_3$) spectrum of **8**.

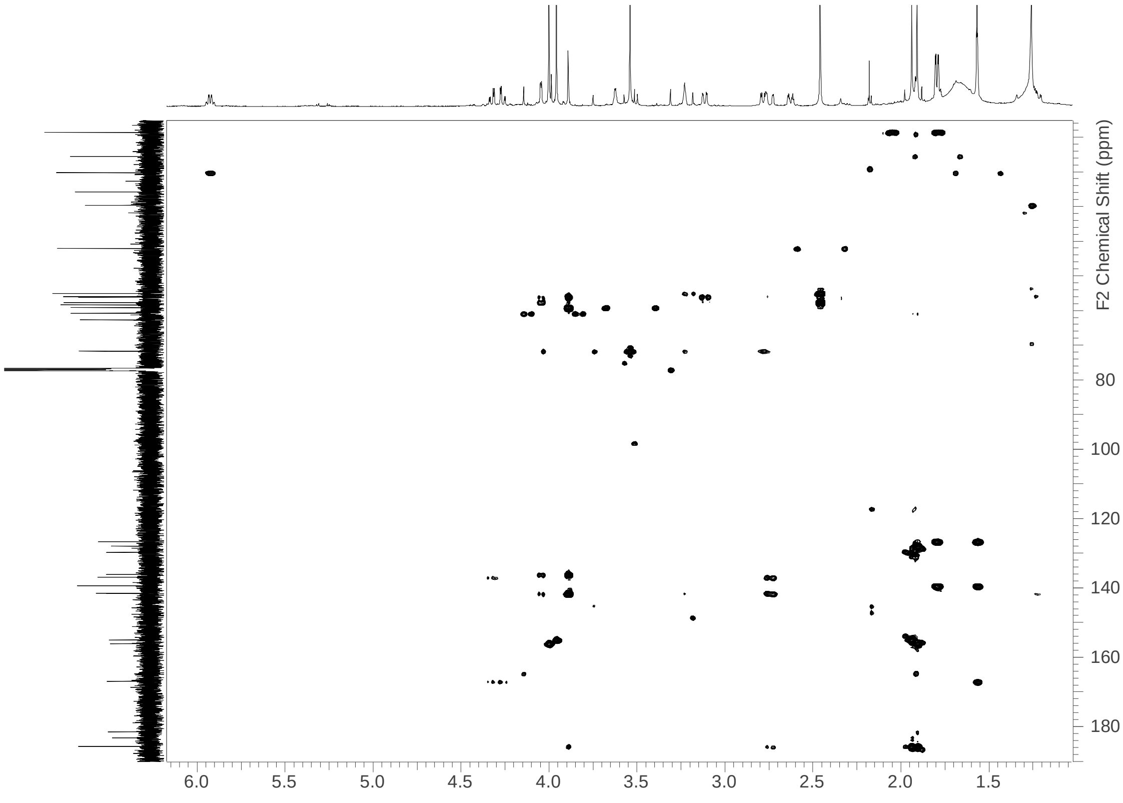

Figure S19. HMBC NMR (500 MHz, $CDCl_3$) spectrum of **8**.

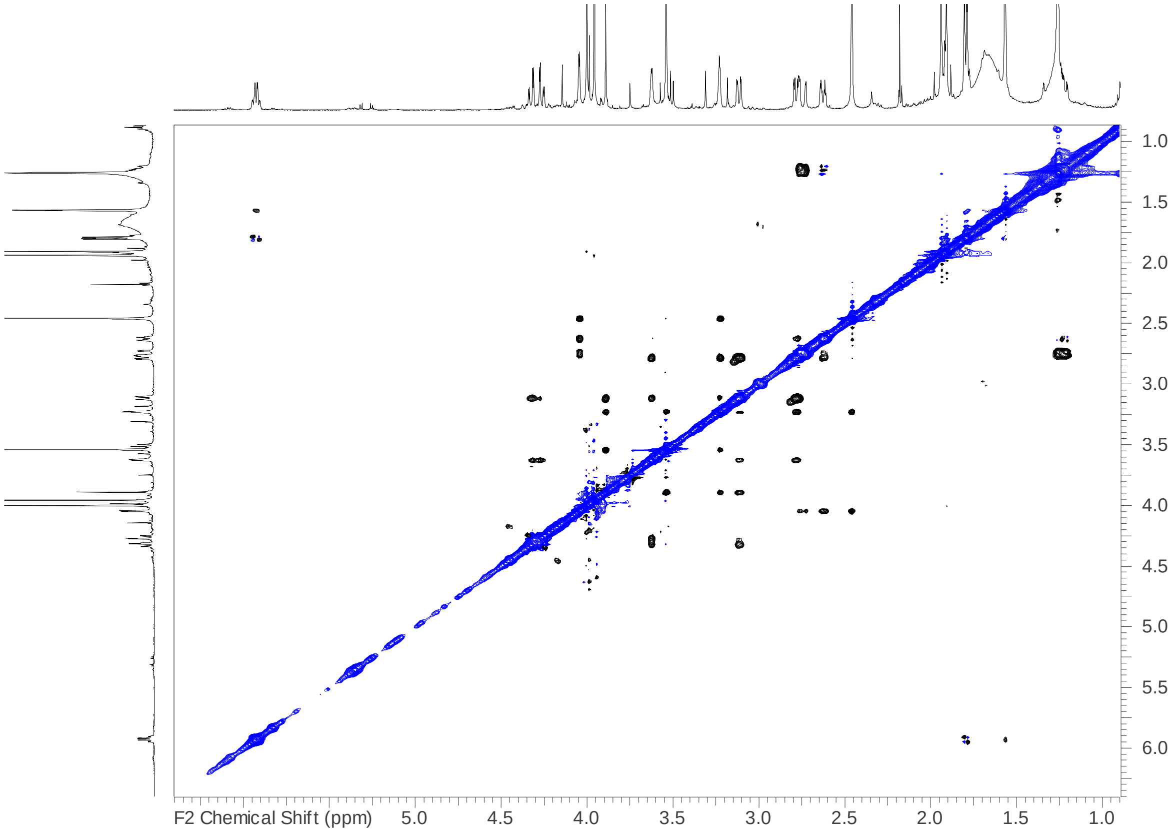

Figure S20. NOESY NMR (500 MHz, $CDCl_3$) spectrum of **8**.

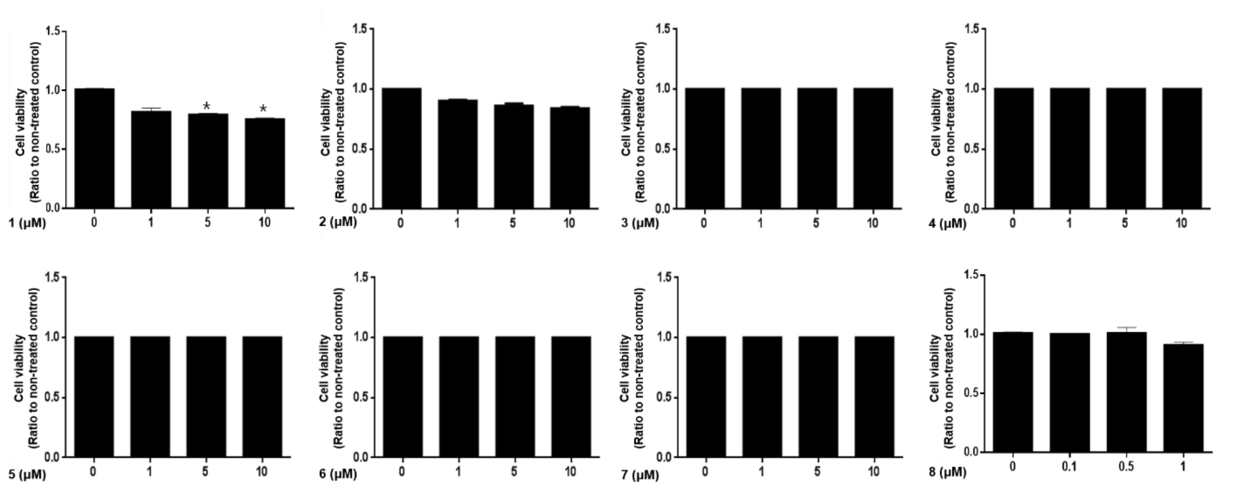

Figure S21: Cytotoxicity of compounds **1~8** against THP-1 cells. THP-1 macrophages were treated with each compound (1-8) at three concentrations indicated. After 24 h of incubation, cell viability was measured using a CCK-8 assay as described in materials and methods. Results are presented as the means ± SDs of triplicate experiments; *$p < 0.05$ compared to non-treated control cells.

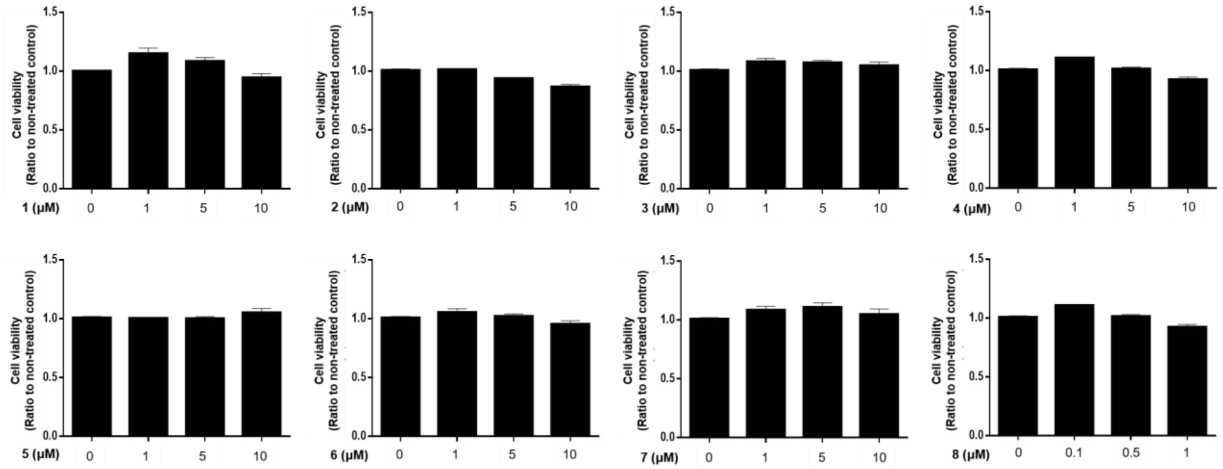

Figure S22: Cytotoxicity of compounds **1~8** against Caco-2 cells. Caco-2 epithelial cells were treated with each compound (1-8) at three concentrations indicated. After 24 h of incubation, cell viability was measured using a CCK-8 assay as described in materials and methods. Results are presented as the means ± SDs of triplicate experiments.

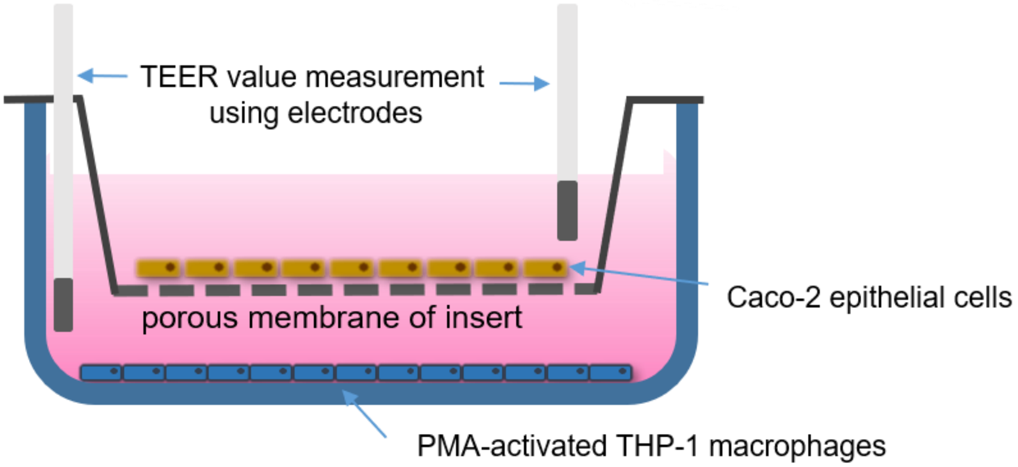

Figure S23: In vitro co-culture system of Caco-2 and THP-1 macrophages

Table S2. Coordinate for the optimized conformer of compound **1**.

Center Number	Atoms	Atomic type	X	Y	Z
1	C	0	-0.67446	2.634854	-0.07909
2	C	0	1.39187	0.84104	0.126022
3	C	0	-0.9777	1.277806	-0.03478
4	C	0	0.668978	3.013855	-0.0239
5	N	0	1.683583	2.145527	0.07913
6	C	0	0.074713	0.351406	0.064985
7	C	0	-0.24241	-1.087272	0.094751
8	C	0	-1.67882	-1.497746	0.045607
9	C	0	-2.71373	-0.618451	-0.04327
10	C	0	-2.41532	0.822146	-0.09279
11	O	0	-3.31192	1.657879	-0.17941
12	C	0	-4.15117	-1.043825	-0.09725
13	O	0	-1.86253	-2.826589	0.090559
14	O	0	0.603697	-1.979409	0.154744
15	C	0	2.594372	-0.058939	0.322516
16	O	0	3.03689	-0.333707	1.415289
17	O	0	3.118615	-0.437844	-0.84919
18	C	0	4.279801	-1.293805	-0.76906
19	H	0	-1.47213	3.365122	-0.15612
20	H	0	0.947774	4.064041	-0.06236
21	H	0	-4.24031	-2.129762	-0.05697
22	H	0	-4.62406	-0.676746	-1.01445
23	H	0	-4.71065	-0.605298	0.736071
24	H	0	-0.97109	-3.227938	0.149854
25	H	0	4.02259	-2.219952	-0.25053

26	H	0	5.08601	-0.785596	-0.23576
27	H	0	4.562039	-1.491612	-1.80214

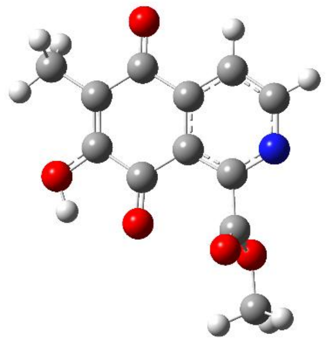

Table S3. Experimental and calculated ^{13}C NMR chemical shifts of compound **1**.

	Experimental (Exp.) data Chemical shifts (ppm)	Calculated (Cald.)Data Shielding shifts (ppm)	Calculated (Cald.) Data Chemical shifts (ppm)	Difference between Exp. and Cald.
1	150.6	27.8983	152.1	-1.5
3	155.6	22.6133	157.1	-1.5
4	120.5	61.8512	119.5	1.0
5	182.8	-5.9330	184.5	-1.7
6	121.9	58.2206	123.5	-1.1
7	153.4	26.0895	153.8	-0.4
8	179.2	-1.8495	180.6	-1.4
9	120.1	62.3536	119.0	1.1
10	139.0	42.4741	138.1	0.9
11	166.6	10.4171	168.8	-2.2
6-CH_3	8.8	175.9468	10.0	-1.2
OCH_3	53.4	131.1725	53.0	0.4

Scaling factor for MPW1PW91/6-311+G(2d,p)//B3LYP/6-31+G(d,p)

- slope : -1.0420
- intercept : 186.3567

(http://cheshirenmr.info/ScalingFactors.htm)
